# Gause's Principle and the Effect of Resource Partitioning on the Dynamical Coexistence of Replicating Templates

**DOI:** 10.1371/journal.pcbi.1003193

**Published:** 2013-08-22

**Authors:** András Szilágyi, István Zachar, Eörs Szathmáry

**Affiliations:** 1 Department of Plant Systematics, Ecology and Theoretical Biology, Institute of Biology, Eötvös University, Budapest, Hungary; 2 Department of Plant Systematics, Ecology and Theoretical Biology, Research Group of Ecology and Theoretical Biology, Eötvös University and The Hungarian Academy of Sciences, Budapest, Hungary; 3 Parmenides Center for the Conceptual Foundations of Science, Munich/Pullach, Germany; University of Texas at Austin, United States of America

## Abstract

Models of competitive template replication, although basic for replicator dynamics and primordial evolution, have not yet taken different sequences explicitly into account, neither have they analyzed the effect of resource partitioning (feeding on different resources) on coexistence. Here we show by analytical and numerical calculations that Gause's principle of competitive exclusion holds for template replicators if resources (nucleotides) affect growth linearly and coexistence is at fixed point attractors. Cases of complementary or homologous pairing between building blocks with parallel or antiparallel strands show no deviation from the rule that the nucleotide compositions of stably coexisting species must be different and there cannot be more coexisting replicator species than nucleotide types. Besides this overlooked mechanism of template coexistence we show also that interesting sequence effects prevail as parts of sequences that are copied earlier affect coexistence more strongly due to the higher concentration of the corresponding replication intermediates. Template and copy always count as one species due their constraint of strict stoichiometric coupling. Stability of fixed-point coexistence tends to decrease with the length of sequences, although this effect is unlikely to be detrimental for sequences below 100 nucleotides. In sum, resource partitioning (niche differentiation) is the default form of competitive coexistence for replicating templates feeding on a cocktail of different nucleotides, as it may have been the case in the RNA world. Our analysis of different pairing and strand orientation schemes is relevant for artificial and potentially astrobiological genetics.

## Author Summary

The dynamical theory of competing templates has not yet taken the effect of sequences explicitly into account. One might think that complementary sequences have very limited competition only. We show that, despite interesting sequence effects, competing template replicators yield to Gause's principle of competitive exclusion so that the number of stably coexisting template species cannot exceed the number of nucleotide species on which they grow, although one of the findings is that plus and minus strands together count as one species. Thus up to four different templates/ribozymes can constitute the first steps to an early, segmented genome: we suggest that other mechanisms build on this baseline mechanism.

## Introduction

Gause (1934) in the Golden Age of theoretical ecology formulated the principle of competitive exclusion, proposing in effect what usually is being referred to as “complete competitors cannot coexist” [1]. Later investigations have confirmed that in stable steady state the number of coexisting species cannot be larger than the number of resources, provided that growth rates depend linearly on resource concentrations and that we look for coexistence at fixed densities [2]–[4]. For maximal coexistence to occur, the 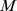 competitors must consume the 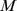 resources in different proportions. Since the seminal experiments of Spiegelman [5] and the deep theoretical insights of Eigen [6], nucleic acid replication kinetics has been under repeated scrutiny. In the “default” model of Eigen with constant total population concentration the fastest replicator (and its associated mutant cloud) wins, consonant with “survival of the fittest”; the tacit assumption being that the competing sequences are complete competitors in the sense of Gause. More detailed investigations of RNA replication kinetics have greatly improved these models, taking into account saturation of the replicase enzyme, asymmetry of plus and minus RNA strands, and replicationally inert double-strand formation [7]–[9]; the latter phenomenon yielding coexistence due to the self-limitation of growth. Von Kiedrowski [10], [11] discovered a somewhat similar phenomenon for his artificial non-enzymatic chemical self-replicators growing parabolically, where self-limitation of growth arises from reversible double-strand formation. Szathmáry and Gladkih [12] showed that the consequential parabolic growth leads to stable dynamical coexistence. Yet none of these models included a detailed analysis of base composition and sequence effects on coexistence. In this paper we remedy this deficiency.

We explicitly take into consideration the concentration of up to four different building blocks (“nucleotides”, with the aim that the model should be general enough to deal with different number of bases and base-pairing modes [13]–[15]) and a large number of competing different sequences, in order (i) to present, at least in part, the missing theory of competing template replicators having different sequences and (ii) to answer the question whether Gause's principle holds for such replicators.

During the forthcoming analysis we deliberately introduce some simplifications. We assume that template replication rates depend on nucleotide concentration linearly (there are no cooperative effects) and that the dynamics of these abiotic resources are not periodically forced, for example. We neglect replicase enzymes and assume that template and replica separate irreversibly upon completion of elongation. The kinetic effects are simplified to the extent that the elongation rate of template polymerization depends only on the identity of the inserted nucleotide and nothing else. We know that this is a crucial simplification but already with this rule different sequences may assume very different kinetic phenotypes. In agreement with this, we neglect secondary and tertiary structures.

The *raison d'être* for these assumptions is that we would like to demonstrate the effect of competition for resources of competing template sequences as simply and clearly as possible. (We note that as mentioned above, irreversible or reversible pair formation can lead to coexistence, and that enzyme saturation leads to linear growth instead of exponential.) We deliberately want to see the dynamics of coexistence under irreversible exponential growth tendency, as a kind of worst case. The effect of the sequence diversity of templates on dynamical coexistence is not trivial. If there are two resources A and B, then it is trivial that sequences of 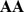 and 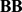 may coexist. But what about 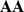 and 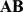
? Are these sufficiently similar for competitive exclusion or sufficiently different for competitive coexistence? And have 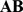 and 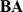 got the same features in competition, or not? This question is relevant since a recent study [16] in the ecological literature indicated that life-history traits of organisms can promote dynamical coexistence on limiting resources beyond the effect of simple resource partitioning. Thus two templates with the same nucleotide composition but substantially different sequences may be regarded as adopting two different life history strategies. As replication proceeds sequentially, templates might be regarded as consuming different resources during different stages of their life histories. What is the effect (if any) of this stage-structure on template coexistence?

Some of the effects that we show in this paper are far from trivial. Our calculations show the effect of resource partitioning on template coexistence and shed more light on early molecular evolution, which surely was affected by sequence effects of template replicators.

## Results

To understand the mechanism of coexistence of template replicators (*sequences*) we formulated the dynamics of polynucleotide replication. Here we only explain the necessary basics of our formalism, for the mathematical model see Models Section, for further details see Text S1. Template replicators are assumed to be single-stranded with double-stranded replication intermediates (as for RNA). As a simplification, metabolism responsible for replication is restricted to the common pool of shared monomers, which are either fed from within (protocells) or from the outside (flow reactor).

A sequence is a single polynucleotide strand of the form 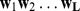 of length 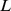 where 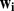 stands for any monomer at the 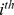 position. As an example, a sequence of monomers in case of RNA could be 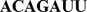 with 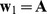, 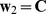, etc. A sequence pair is a double-stranded polynucleotide molecule. It can be represented by only one of its strands as it defines the complement strand unambiguously (note that we do not deal with strand separation and treat the two strands as separate sequences). For example the 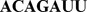 sequence defines its complementary pair 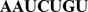. An intermediate complex is a complete sequence and its incompletely built complementary sequence during the duplication phase. For example, one intermediate complex of the above RNA sequence pair is:
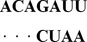



An 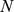-*group of sequence pairs* consists of 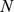 such sequence pairs. Often members of a group are represented by one sequence of each pair for sake of simplicity. Concerning the dynamics, for each type of monomer we introduce a specific rate constant that defines the speed of elongation of the sequence; also for each monomer type and for each intermediate there is a specific degradation rate constant.

**Figure 1:**
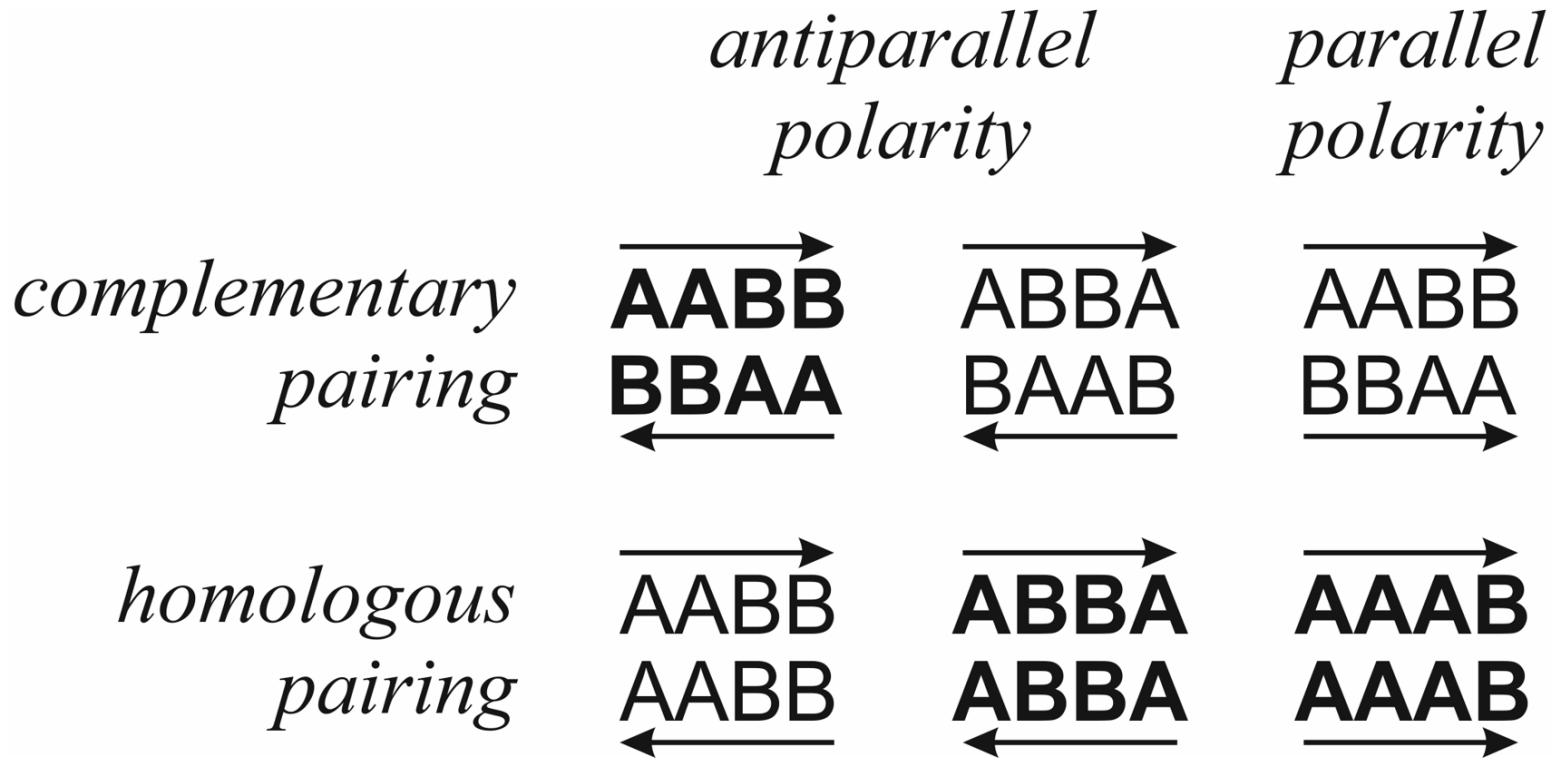
Template and copy are either different (thin) or identical (bold) for complementary (top) and homologous pairing (bottom) due to strand polarity. Reverse (top left) and direct palindromes (bottom middle) yield two identical templates with complementary and homologous pairing, respectively, just like homologous pairing with parallel polarity. Note, that in case of reverse palindromes, it is not necessary for the sequence itself to be palindromic to make the two strands identical. Cases of complementary pairing with antiparallel polarity (top left and middle) and homologous pairing with parallel polarity (bottom right) are discussed in the main text; homologous pairing with antiparallel polarity (bottom left and middle) is discussed in Text S1. The remaining case (top right) is not discussed here.

First, we investigate the more realistic but also more complex systems that can only be solved numerically and later we gradually traverse to simplified systems that can be handled fully analytically. Such systems, though simplified, provide powerful rules about the mechanisms of coexistence which still can be translated and applied to the realistic cases. Accordingly, first we numerically analyze the complementary replication of templates corresponding RNA replication. Second, we deal with the simpler homologous replication where monomers pair with identical types (non-complementary base-pairing); we also introduce parallel strand polarity as opposed to antiparallel polarity (like in case of RNA replication). The difference between complementary and non-complementary pairing and parallel and antiparallel strand polarity is given in Fig. 1. Third, as a further simplification of the previous system, we assume uniform degradation rates for replication intermediates and even identical elongation rates for the different monomer types to obtain analytical results.

### Complementary replication

The following model of template replication models RNA replication, dealing with 4 monomers, complementary pairing and antiparallel strand polarity. We have investigated the coexistence of 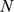 groups of sequence pairs of length 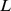. As the sequence space is huge (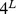), consequently, the Cartesian product of the sequence space yielding all possible combinations of 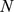 sequence pairs is even more huge (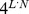). Therefore it is usually impossible to investigate the coexistence of all possible combinations of sequences. Instead, we used a reasonably large sample of the full combined space to estimate the probability and stability of coexisting sequences over four different monomers. We have investigated sequences of length 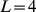, as this is the maximum length for which the space could be reasonably analyzed. Our analysis was performed using the following two methods (for parameters, see Text S1):

- M1 With a given set of parameters (elongation constants 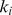, degradation rates 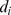 and 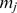) we computed the fraction 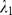 of coexisting sequence groups of the total combined sequence space with different numbers of complementary pairs (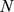). In case of a large sequence space, only a random subset was used. In case of coexistence, we examined the local stability of this coexistence.
- M2 We have investigated the coexistence for each sampled sequence group for different numbers of complementary pairs (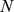) using 1000 different random degradation rate sets. We have assessed the ratio 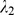 of coexisting sequences.

The results of the analysis of coexistence of 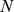 complementary pairs of sequences of length 4 can be seen in Tables 1 and 2 (methods M1 and M2, respectively). Accordingly, we can state that the increasing number of complementary pairs reduces the probability *and* stability of coexistence and that on four different monomers a maximum of four different sequence pairs could possibly coexist. Thus Gause's principle (against first intuition) limits the number of *sequence pairs* instead of *individual sequences* because of the dynamical coupling between the template and its complement (the plus-minus ensamble behaves like a single replicator, see [6], [17]). In the intermediate case of non-complementary pairing with antiparallel polarity, it is still the number of sequence pairs that limits coexistence (and not individual sequences), though due to a better partitioning of resources coexistence is more probable on average (for details, see Text S1).

**Table 1:** Analysis of coexistence of 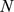 complementary pairs of sequences of length 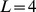 according to method M1.

*N*	Number of scanned seq.s	Fraction of coexistence (*λ*_1_)	Average of leading eigenvalues
2	4^8^ (100%)	55.5% (S)	−0.0401
3	10^6^ (5.96%)	11.1% (S)	−0.00198
4	10^6^ (0.023%)	0.83% (S)	−0.000653
5	10^7^ (0.0009%)	0%	n/a

Second column shows the number of scanned sequences (and the amount as a fraction of the whole combined sequence space). The third column shows the fraction of coexisting sequences in the scanned domain, i.e. the probability of coexistence of random sequence groups of size 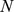 with a given parameter set. (S) indicates that all cases of coexistence are locally asymptotically stable. The fourth column shows the average of the leading eigenvalues (if there is coexistence) as a measure of stability. For parameters, see Text S1.

**Table 2:** Analysis of coexistence of 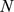 complementary pairs of length 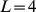 according to method M2.

*N*	Number of scanned seq.s	Fraction of coexistence (*λ*_2_)
2	4^8^ (100%)	49.40%
3	4^8^ (0.4%)	6.92%
4	4^8^ (0.0015%)	0.36%
5	4^8^ (6⋅10^−6^%)	n/a

Second column shows the number of scanned sequences (and the amount as a fraction of the combined sequence space). The third column shows the fraction of coexisting sequences averaged over the scanned sequence groups and over 1000 random degradation rate sets for intermediates (for parameters, see Text S1).

**Figure 2:**
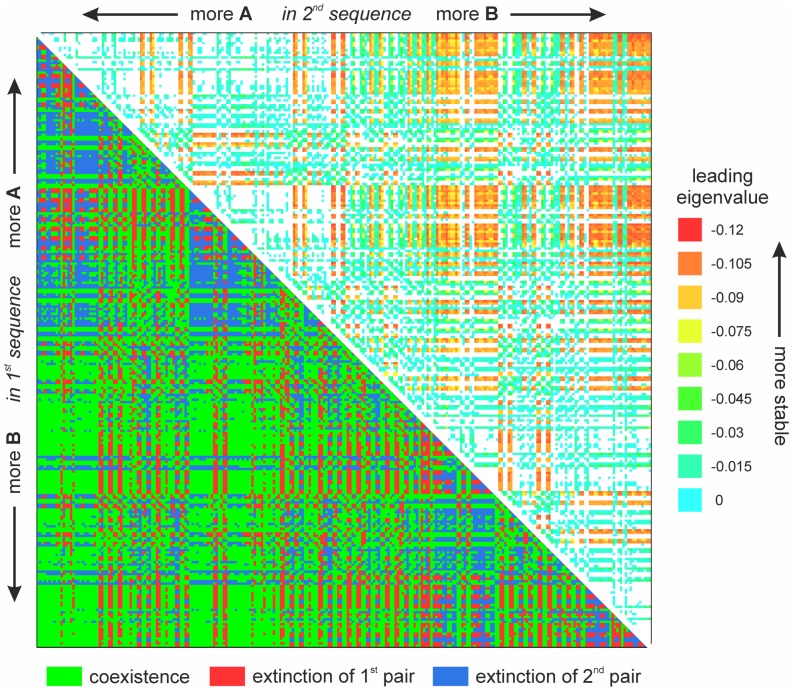
Split plot of the coexistence of two complementary sequence pairs with antiparallel strand polarity (4 sequences per pair) of length 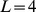. Lower left half: coexistence is marked by green, extinction of the first sequence pair by red and extinction of the second sequence pair by blue. Upper right half: stability of coexistence according to the leading eigenvalue (red indicates more stable, blue indicates less stable coexistence, white indicates extinction of one of the sequence pairs). The upper triangle shows the stability measures of the sequences pairs from the lower one (mirrored and rotated 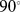). From the point of view of coexistence two pairs (e.g. 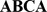-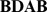, 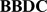-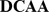) and their reverse (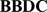-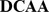, 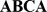-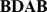) are not fully equivalent. The reason for this is that the degradation rates are assigned to sequences, always in the same order within a set (this means that the same rates are assigned to e.g. 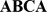 and 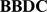 in the two cases, respectively). Despite this difference the plot is almost symmetrical since degradation rates are taken from a narrow distribution. For details, see main text, for parameters, see Text S1.

Coexistence can be visualized in case of two coexisting sequence pairs of length 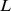 in two dimensions: each cell of a 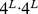 matrix represents a certain combination of two pairs, and is labeled by the first sequence of the first pair (rows) and the first sequence of the second pair (columns). Sequences are ordered according to standard lexicographic ordering along the horizontal and vertical axes (see Fig. 2). The higher probability and mean stability of coexistence in the bottom corner correspond to the “niche partitioning”: first sequence consumes more 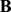, while the second sequence consumes more 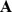.

Our model is applicable for any sequence length 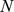, predicting whether there is coexistence for any combination of sequences. However, for larger values of 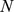 the full combined sequence space is enormous therefore it is impossible to perform exhaustive search for all coexisting cases. By brute force search we have found some illustrative examples, according to Method M2 (for details see Text S1). Our results (tested up to 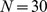) show that 8 sequences (4 sequence pairs) can stably coexist (linear asymptotic stability was explicitly tested). There is no theoretical (but computational) objection to applying this model for even longer sequences.

### Non-complementary (homologous) replication

In this section we deal with a simplified system: we restrict our attention to homologous base pairing ignoring the polarity of strands (see Fig. 1), allowing only two monomer types (see the extended model of four monomers in the Text S1). Due to homologous pairing of monomers and the lack of different polarities of the strands, template and copy are identical, thus a pair of pairs consists of a total of two different sequences. Intermediates of the first sequence (pair) are denoted as 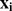, intermediates of the second are denoted by 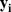, their concentrations by 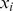 and 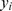 (as were introduced previously), monomers are denoted by 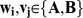 and their concentrations by 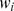 and 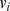, (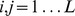). Note that results in this section hold for all cases when template and copy are identical: this happens not only in the case of homologous pairing with parallel orientation, but also for some exotic cases of antiparallel strands, like palindromic sequences of homologous pairing and reverse-palindromic sequences of complementary pairing (Fig. 1). We briefly note that such palindromes are not likely to coexist any more than non-palindromic sequence pairs.

#### Non-uniform degradation rates

As there is no complementary pairing or polarity, any sequence pair consists of two identical sequences, thus the maximum number of coexisting species on two different resources is expected to be two. With the applied restrictions, the system still remains overly complex for an analytical approach (see the next two sections for an analytically tractable simplification). According to our exhaustive numerical results, we have found no case where more than two sequences could coexist, supporting our hypothesis. We have numerically integrated ODE systems until convergence or extinction of one of the sequences. The numerical methods and routines are the same as before. We have analyzed the coexistence of two sequences in two different ways (for parameters see Text S1):

- M3 With a given set of parameters (elongation constants 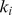, degradation rates 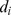 and 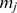) we computed the fraction 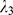 of coexisting sequence pairs of the total combined sequence pair space at different sequence lengths (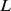). In case of coexistence, we examined the local stability.
- M4 We have investigated the coexistence for each sampled sequence pair using 1000 different random degradation rate sets. We have assessed the ratio 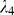 of coexisting sequences as a function of the length 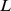 of the sequences and the stability of the coexistence.

First, the results of our investigations according to method M3 can be found in Table 3. It can be observed that approximately half of the sequences coexist, independent of the sequence length, while increasing length decreases the mean stability (defined as the mean of leading eigenvalues). We have always found coexistence to be locally asymptotically stable.

**Table 3:** Analysis of coexistence of two sequences of length 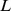 according to method M3.

*L*	Number of scanned seq.s	Fraction of coexistence (*λ*_3_)	Average of leading eigenvalues
3	2^6^ (100%)	50.0% (S)	−0.0811
4	2^8^ (100%)	43.4% (S)	−0.0611
5	2^10^ (100%)	50.3% (S)	−0.0598
6	2^12^ (100%)	46.1% (S)	−0.0513
7	2^14^ (100%)	49.5% (S)	−0.0462
8	2^16^ (100%)	47.8% (S)	−0.0412
9	2^18^ (100%)	48.9% (S)	−0.0355

Second column shows the number of scanned sequences (the whole combined sequence space for all investigated 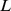). The third column shows the fraction of coexisting sequences, i.e. the probability of coexistence of two sequences with a given parameter set (see Text S1). (S) indicates that all cases of coexistence are locally asymptotically stable. The fourth column shows the average of the leading eigenvalues (if coexistence exists) as a measure of stability.

Second, we have investigated the system according to method M4. The results characterizing the coexistence depending on 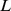 are in Table 4. Approximately 50% of the randomly parameterized sequence pairs can coexist (independently of sequence length) and the (average) stability of the coexistence is slowly decreasing with increasing length.

**Table 4:** Analysis of coexistence of two sequences of length 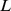 according to method M4.

*L*	Number of scanned seq.s	Fraction of coexistence (*λ*_4_)
3	2^6^ (100%)	49.98%
4	2^8^ (100%)	42.81%
5	2^10^ (100%)	47.06%
6	2^12^ (100%)	43.15%
7	2^14^ (100%)	44.88%
8	2^14^ (25%)	43.24%
9	2^14^ (6.25%)	43.68%

Second column shows the number of scanned sequences (and the amount as a fraction of the whole sequence space). The third column shows the fraction of coexisting sequences averaged over the scanned sequence pairs and the 1000 random parameter sets. For parameters, see Text S1.

**Figure 3:**
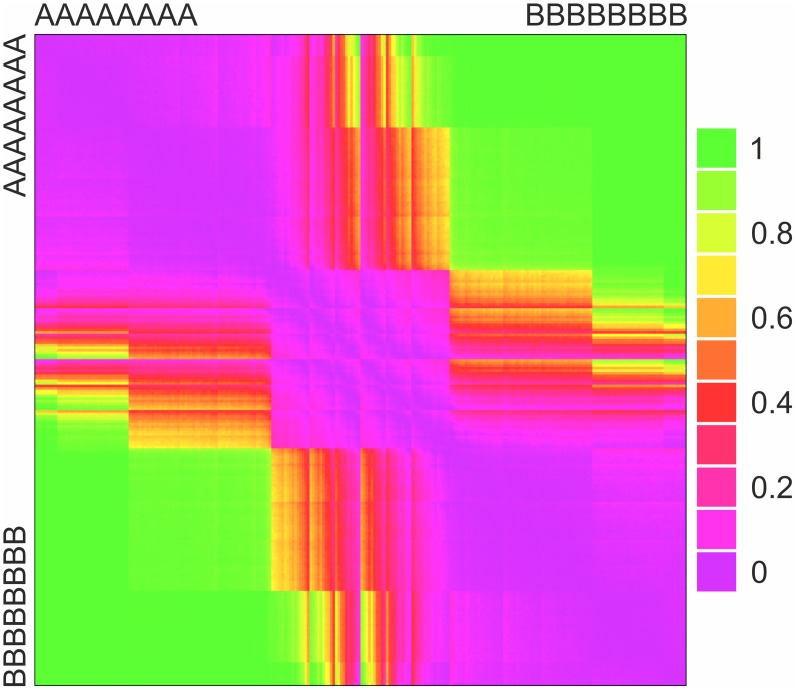
Probability of coexistence in case of non-uniform degradation rates and homologous pairing (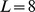). Each cell is an average of numerical results over 1000 random parameter setups. Purple indicates highly improbable coexistence, green indicates likely coexistence. Sequences are arranged along the axes first according to Hamming distance and secondly according to lexicographic ordering (increasing 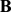 content towards bottom and right). For parameters, see Text S1.

**Figure 4:**
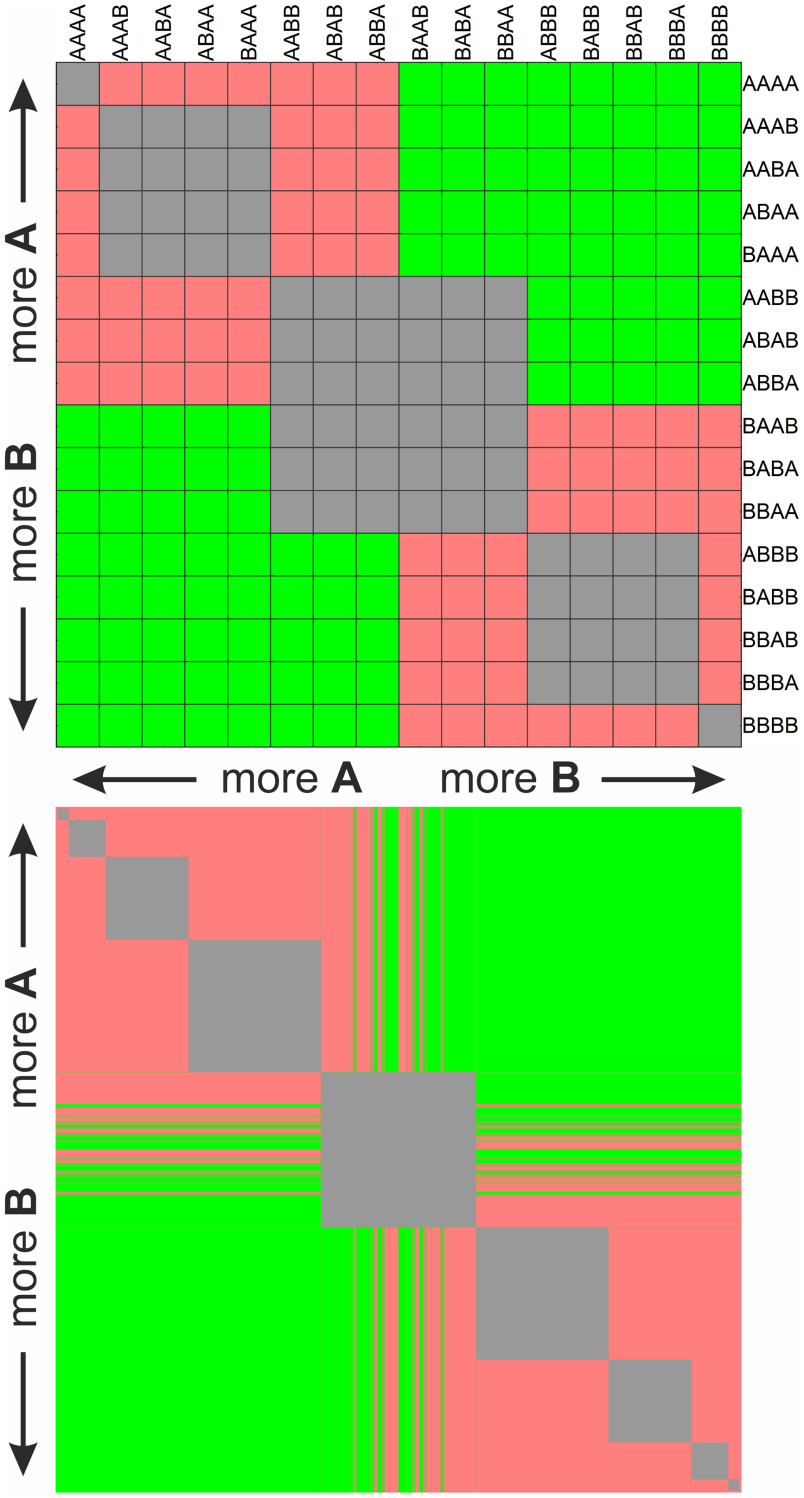
Coexistence plots of pairs of double-stranded sequences of length 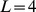 (upper panel) and 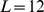 (lower panel) using two monomers (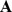, 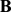) in case of uniform degradation and identical elongation rate constants and non-complementary pairing. The green indicates stable coexistence, grey indicates structurally unstable coexistence, i.e. compositional identity (no coexistence in a biological sense), while pink indicates that there is no coexistence possible. Sequences along the axes are arranged first according to Hamming distance and secondly according to lexicographic ordering from more 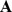 (top and left) to more 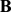 (bottom and right). For parameters, see Text S1.

For two sequences, it is possible to visualize coexistence results over the random parameter sets. As Fig. 3 shows, coexistence is more likely if paired sequences contain different amounts of 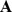 and 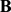 (lower left and upper right corners). Purple boxes along the main diagonal indicate improbable coexistence, corresponding to *compositionally identical* sequences that is, 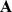
∶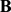 ratios for the two sequences are identical (cf. the grey areas of Fig. 4).

#### Uniform degradation rates

Uniform degradation rates of sequence intermediates allow for a completely analytic approach. With this simplification, we can compute the stable steady state concentrations of intermediates and monomers analytically. We managed to simplify the criteria of coexistence to a pair of simple inequalities in terms of the number and position of different monomers in the sequences (Eq. (14)). Our results indicate that more than two sequences cannot coexist in a structurally stable way on two different monomers. Generally, it is true that a maximum of 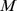 different sequences can coexist on 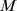 different monomers for details, see Text S1. We have investigated the local asymptotic stability of coexistence. For different sequence lengths (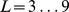) we have examined the whole sequence space (excluding compositionally identical cases). Numerical results indicate that solutions are locally asymptotically stable (parameters are the same as in method M3).

The above criteria of coexistence only hold for compositionally non-identical sequences. In case of compositional identity, we have demonstrated numerically (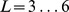, for the whole sequence space) that the coexistence is neutral. All stable steady state concentrations depend on the initial conditions (the leading eigenvalue of the Jacobian being zero). From a biological point of view, coexistence of compositionally identical sequences is *structurally unstable*, as any perturbation of the degradation rates disrupts coexistence, thus practically there is no coexistence. (With non-uniform degradation rates, compositionally identical pairs can stably coexist with very low probability; cf. Fig. 3, main diagonal blocks) In the following calculation (and later, if not indicated otherwise), we have ignored compositionally identical sequence pairs.

We have demonstrated numerically, that more than two arbitrary sequences are able to coexist, though in a structurally unstable way: any perturbation of the degradation rates destroys coexistence, rendering the system to a simpler one where only one or two sequences coexist on the two monomers. Thus Gause's principle is not violated.

#### Uniform degradation rates and identical rate constants

As a further simplification, here we assume identical rate constants, which allows a fully analytical approach. In this case, the presence (or lack) of coexistence of sequences can be deduced right from the order of monomers of the sequences according to a very simple rule (Eq. (15)). The rule provides a deeper understanding of coexistence by revealing a further effect additional to simple resource partitioning. According to the rule, the criteria of coexistence in case of shorter sequences (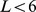) leads to the exclusion principle of Gause. Thus two sequences coexist if they have different limiting resources, that is if one has more 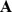 while the other has more 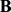, as in “niche-segregation” (compact green areas in Fig. 4, upper panel). Compositionally identical sequences result in neutral stability (and structural instability against perturbation of degradation rates), see gray blocks in Fig. 4.

However, for longer sequences (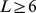), this is only generally true, and it becomes possible to seemingly violate the principle, as there are exceptions depending on the position of monomers that allow for coexistence even when sequences seemingly feed on the same limiting resource. For example, two sequences can coexist if both have e.g. 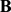 majority, provided one of the sequences has most of the **A**-s in its head; the latter behaves as having 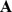-majority. The coexistence condition directly (Eq. (15)) specifies the required amount and position of **A**-s in the head to yield coexistence (for example, 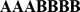 and 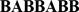 coexist, seemingly violating Gause's principle). Therefore, it is not just the composition of the sequences but the order of monomers too that affects coexistence, thus an explicit sequence effect is present. The number of these “irregular” coexistent cases increases with sequence length: this finding corresponds to the stage-structure effect found in theoretical ecology [16]. Note, however, that Gause's principle in the end holds since there cannot be more sequence pairs than the number of limiting resources.

To sum up, according to the coexistence condition (Eq. (15)), the front part of the sequence (*head*) weighs more concerning the coexistence than the rear (*tail*). This is because during replication, earlier intermediates are present in larger concentrations than intermediates closer to the final step of the replication (for details, see Text S1). Consequently, monomers included earlier (i.e. into the head) are needed in higher concentrations than monomers incorporated into the tail. Thus the head influences competition more strongly than the tail.

A special but analytically tractable case is when the head and tail are homogeneous blocks of identical monomers. If there are at least
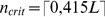
(1)


**A**-s in a homogeneous block in the head, the sequence behaves as having 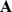-majority, and can coexist with sequences explicitly having **B**-majority (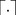
denotes the closest integer being larger or equal; the exact value of this constant is: 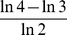, for proof, see Text S1). For the previous example, 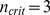, the first sequence behaves as having **A**-majority and it coexists with the second sequence, though both have 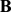-majority.

If we visualize the coexistence of such pairs where each sequence in the sequence space is listed along the horizontal and vertical axes in the same order, examples of irregular coexistence can be found as the green lines at the borders of bottom left (“less 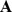 – more 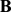
”) and upper right (“more 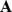 – less 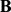
”) domains (Fig. 4, bottom panel). The longer the sequence gets the more obvious is the effect and the irregularity: the borders of the two main domains get more fractured. However, no new coexisting pairs appear, due to the symmetry, since each irregularly coexisting case prevents the regular coexistence of its symmetric pair. However, the number of irregularly coexisting pairs will increase, as the homogeneous blocks in the “head” of the sequence (the first 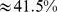 part) allow for more irregular coexistence (it is supposed that for long enough sequences it is not necessary for the head-block to be fully homogeneous, i.e. to exclusively contain one monomer only). According to Eq. (15), for 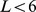, 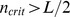, irregular coexistence is not possible as head-monomers will be in explicit majority (compare internal structures of panels of Fig. 4).

## Discussion

In this paper we have provided the foundations of the hitherto missing theory of template replication where replication intermediates and different sequences are explicitly taken into account. Under the assumption of fixed stable steady state densities for resources and competitors Gause's principle [1] fully rules over replicator dynamics: coexistence of more replicators than the number of limiting resources (nucleotides) is not asymptotically stable. We have found, however, that template and copy (or plus and minus strands) count as one replicator, since they are stoichiometrically coupled.

We have found cases of coexistence where Gause's principle seems to be violated in that two sequences can coexist with exactly the same nucleotide composition but adequately different sequences: this is a version of the stage-structure effect on coexistence found in theoretical ecology [16]. The part of a sequence that is replicated earlier has a stronger effect than that replicated later, since replication intermediates corresponding to positions in the front are more abundant, hence they influence competitive dynamics more strongly. We have demonstrated the trend that the stability of coexistence in terms of the leading eigenvalue decreases with sequence length. This may be considered bad news; however we should not forget that a good share of ribozymes [18] and aptamers [19] is smaller than 100 nucleotides, for which one still would get acceptable local stability values. (Note that the smallest known ribozyme consists of 5 nucleotides [20].)

The relevance of our findings can be questioned on the grounds that ribozyme replicators should have been longer than considered in this paper. This objection partially loses force if one considers known ribozyme sizes and the early constraints on replication. We discuss these issues in turn. The smallest ribozymes known are: (1) the trinculeotide 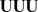 that catalyses metal ion-dependent cleavage of RNA [21], (2) the pentanucleotide 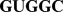 promoting peptide bond formation [22], and (3) the 19 nucleotides long minimalized hammerhead ribozyme for RNA cleavage [23]. Note that these ribozymes are devoid of complex secondary structures that would significantly alter their potential replication kinetics. Nevertheless, Yarus notes that the stable conformation of a cavity formed by the single-stranded overhang beyond the three base pair formed between enzyme and substrates seems to be essential for catalysis [22]. Regarding the replication issue, it is generally believed that small RNA replicators preceded long ones, partly supported by the non-enzymatic replication in the von Kiedrowski experiments [10]. In fact, the production of a generalized replicase ribozyme that could replicate long RNA-s is an unsolved problem. This prompted Ellington [24] to suggest a collectively autocatalytic set consisting of a modest replicase and a ligase. In such a system only small fragments would be replicated, followed by ligation to yield the longer ribozyme structures. Noteworthy in this regard is the case of the collectively autocatalytic, ligating set of Lehman [25], in which fragments of lenghts 43, 65, 55 and 52 are used as pieces in the assembly. It remains to be seen whether these fragments would stably coexist when replicated, using the right combination of the resource and structure fitness landscapes. Of course, we might find in the future ribozymes that could be assembled from even smaller pieces; for such cases our theory would almost immediately apply. In any case, we predict that dynamical coexistence of small, functionally important RNA replicators will be demonstrated in the near future.

Mechanisms for template coexistence have been in the focus of models of primordial replicator evolution (cf. [6], [26]). Here we have shown that up to four replicator pairs (plus and minus sequences) can stably coexist in the same environment without any special coupling. Thus we argue that for any special theory showing that 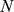 different template replicators can coexist one might find that in effect up to 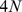 different replicators may coexist without explicit representation of the four nucleotides as resources. This calls for further investigations.

Recently there has been an upsurge in interest in exo/astrobiology. It is in this context that we have deliberately presented results for homologous pairing also, even with parallel orientation of the strands. Although such configurations are not unheard of even in our world, we wanted to see how such features would in general affect dynamical coexistence of template replicators.

We have obtained the fitness landscapes through a distribution of elongation and degradation rates. The main reason behind this is tractability: although the 2D structures as phenotypes of RNA molecules can be calculated for most cases, this does not automatically yield phenotypes in terms of replication rates. We are temporarily satisfied with the phenotype richness that our local rules provided (see Fig. S3). What is more, we predict that the main finding that Gause rules over competitive coexistence of template replicators in stable steady state would not be violated even with more complex fitness landscapes.

## Models

### Reaction and dynamics

In each experiment, we integrated the system of 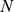 sequence pairs (Eqs. (5)–(9) extended with the dynamics of the rest 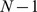 pairs) until convergence (when the difference of the concentrations of any two intermediates at two successive time steps is less than 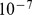) or until extinction (if the concentration of an intermediate is less than 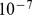 the corresponding sequences pair is treated as extinct). We are interested in how many sequence pairs can coexist maximally on four different monomers. According to Gause's principle, one would expect a maximum of two sequence pairs to coexist, as that yields four different sequences. Since members of the pairs are stoichiometrically coupled, this should affect the dynamics, allowing different mechanisms of coexistence.

Let us introduce two complementary sequences:
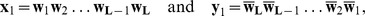
where 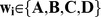 is the 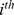 is the type of the monomer at position 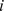. Since 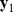 is the complement of 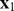, the overbar denotes the complementing monomer pair (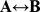 and 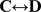, thus 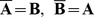, etc.). Replication of the sequences happens as 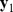 builds up stepwise along 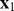. Using the notation above, the intermediate complexes during replication are:
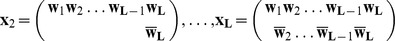


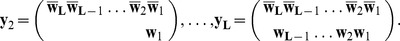
When the new copy is completed along the other template, the two strands separate instantaneously yielding 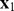 and 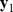. The schema of the reactions is as follows. Equations (2), (3) and (4) correspond to the replication of 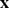 and 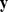 strands and the generation/degradation of components, respectively.
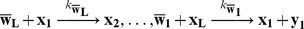
(2)

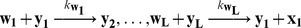
(3)

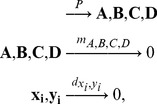
(4)where 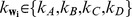 is the elongation rate constant for the given monomer at position 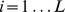, 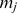 and 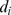 denote degradation rates for the 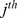 type of monomer and for the 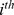 species. The corresponding dynamics of the intermediates is as follows (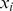, 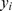 denotes concentration of 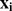 and 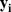, respectively, 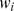 denotes the concentration of the 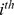 monomer in the sequence, overbar denotes the complementing pair 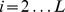):
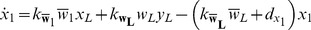
(5)

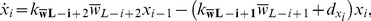
(6)

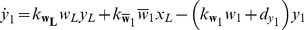
(7)

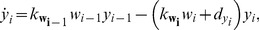
(8)and the dynamics of the monomer 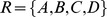, where 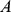 denotes the concentration of monomer 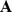, etc. is:
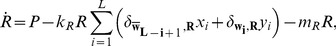
(9)where 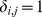 if 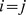, otherwise 0 (Kronecker delta).

The extension of the dynamics for more sequence pairs (i.e., to more than one copy and template) is straightforward. The dynamics of the intermediates is independent for each pair and the dynamics of the monomers provides the coupling between the equations of different pairs of sequences. Because of the cross-coupling of equations, no analytical solution was found (some analytical results will be presented for simplified cases). For the numerical integration of the ODE system to find steady-state solutions we have used the CVODE code from the SUNDIALS project of the Lawrence Livermore National Laboratory [27].

### Non-complementary replication and uniform degradation: An analytical approach

Uniform degradation rates of sequence intermediates allow for a completely analytic approach. For the positivity test of concentrations, we introduce the following notation for the constants of the power sum of 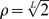:
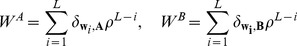
(10)

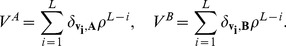
(11)The solution of the dynamics of the non-complementary pairing system with uniform degradation rates for intermediates and monomers (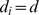 and 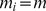, respectively, for all 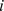) provides the concentrations of the last intermediates of 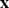 and 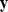 :
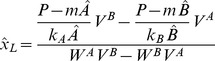
(12)

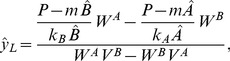
(13)where 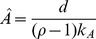 and 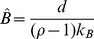 are the stable steady state monomer concentrations (for detailed derivation, see Text S1).

Let us assume that influx can counter degradation. In this case the condition of coexistence (
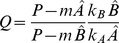
):
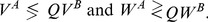
(14)To sum up, coexistence is possible if 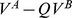 and 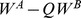 are of *different signs*.

If the two elongation rate constants of the two monomers are *identical* (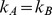) the parameter 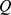 equals 1, thus the simple criteria of coexistence are the following:
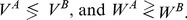
(15)


For example, sequences 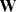 = 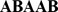 and 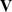 = 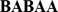 are not coexisting (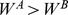 and 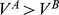), according to Gause's principle, as for both sequences 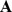 is the limiting resource for which they compete. On the other hand, 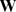 = 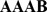 and 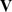 = 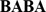 are coexisting (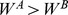 and 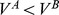).

Sequences 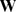 = 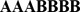 and 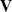 = 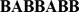 demonstrate an example of irregular coexistence seemingly violating Gause's principle, as both have 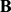-majority. Though, according to Eq. (15), (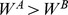 and 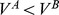), and thus 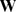 behaves as having 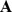-majority.

## Supporting Information

Figure S1
**Split plot of the coexistence of two non-complementary sequence pairs with antiparallel strand polarity (4 sequences per pair) of length** 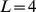 **.** Lower left half: coexistence is marked by green, extinction of the first sequence pair by red and extinction of the second sequence pair by blue. Upper right half: stability of coexistence according to the leading eigenvalue (red indicates more stable, blue indicates less stable coexistence, white indicates extinction of one of the sequence pairs). The upper triangle shows the stability measures of the sequences pairs from the lower one (mirrored and rotated 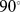). From the point of view of coexistence two pairs (e.g. 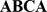-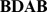, 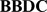-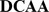) and their reverse (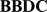-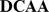, 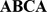-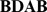) are not fully equivalent. The reason for this is that the degradation rates are assigned to sequences, always in the same order within a set (this means that the same rates are assigned to e.g. 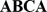 and 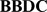 in the two cases, respectively). Despite this difference the plot is almost symmetrical since degradation rates are taken from a narrow distribution. The parameters are the same as in Fig. 2, for details, see the first section of Text S1.(TIF)

Figure S2
**Coexistence plots of pairs of double-stranded sequences of length** 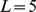 **(upper panel) and** 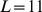 **(lower panel) using two monomers (** 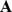 **,** 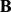 **) in case of uniform degradation and identical elongation rate constants and non-complementary pairing.** The green indicates stable coexistence, grey indicates structurally unstable coexistence, i.e. compositional identity (no coexistence in a biological sense), while pink indicates that there is no coexistence possible. Sequences along the axes are arranged first according to Hamming distance and secondly according to lexicographic ordering from more 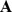 (top and left) to more 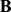 (bottom and right). The parameters are the same as in Fig. 4, for details, see the first section of Text S1.(TIF)

Figure S3
**Correlation plot of the fitness landscape as a function of Hamming distances for sequence pairs of length** 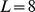 **.**
(TIF)

Table S1
**Analysis of coexistence of** 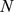 **non-complementary pairs of sequences of length** 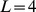 **according to method M1.** Second column shows the number of scanned sequences (and the amount as a fraction of the whole combined sequence space). The third column shows the fraction of coexisting sequences in the scanned domain, i.e. the probability of coexistence of random sequence groups of size 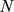 with a given parameter set. (S) indicates that all cases of coexistence are locally asymptotically stable. The fourth column shows the average of the leading eigenvalues (if there is coexistence) as a measure of stability.(XLS)

Table S2
**Analysis of coexistence of** 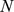 **non-complementary pairs of length** 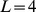 **according to method M2.** Second column shows the number of scanned sequences (and the amount as a fraction of the combined sequence space). The third column shows the fraction of coexisting sequences averaged over the scanned sequence groups and over 1000 random degradation rate sets for intermediates (for parameters, see previous section of Text S1).(XLS)

Table S3
**Examples of coexistence for** 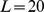 **.**
(XLS)

Table S4
**Examples of coexistence for** 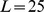 **.**
(XLS)

Table S5
**Examples of coexistence for** 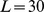 **.**
(XLS)

Text S1 **Supporting text with sections on 1) Parameters for methods M1, M2, M3 and M4; 2) Analysis of non-complementary pairing with antiparallel polarity; 3) Analysis and analytical results of non-complementary pairing and uniform degradation rates; 4) Proofs; 5) Discussion of the fitness landscape; 6) Examples of coexistence of longer sequences.**
(PDF)
