# Supplementary figures and images for: Gause's Principle and the Effect of Resource Partitioning on the Dynamical Coexistence of Replicating Templates
Source: PLoS Comput Biol. 2013 Aug 22;9(8):e1003193. doi: 10.1371/journal.pcbi.1003193 (PMC3749944; doi:10.1371/journal.pcbi.1003193)

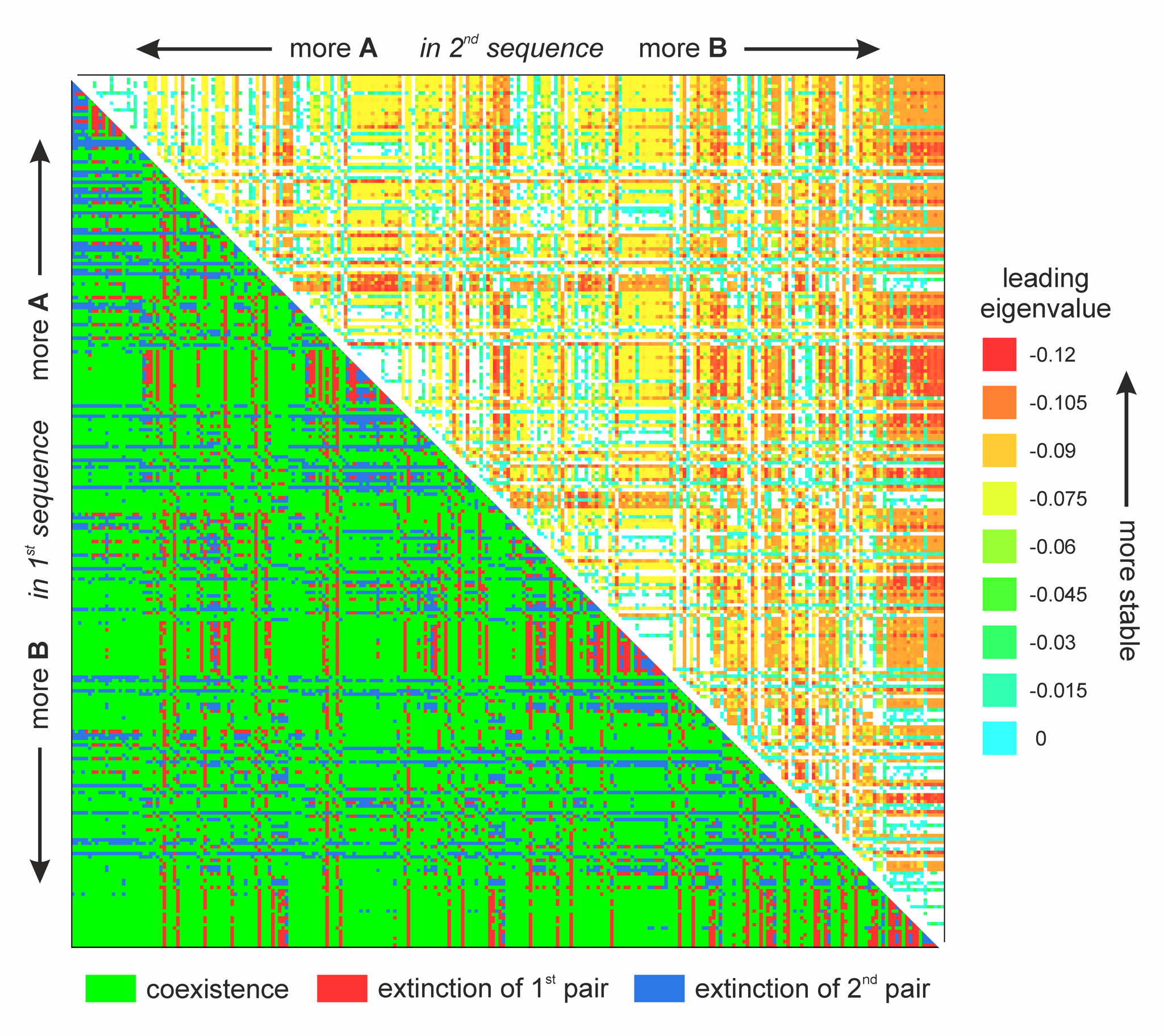

Supplement: Figure S1 — Split plot of the coexistence of two non-complementary sequence pairs with antiparallel strand polarity (4 sequences per pair) of length . Lower left half: coexistence is marked by green, extinction of the first sequence pair by red and extinction of the second sequence pair by blue. Upper right half: stability of coexistence according to the leading eigenvalue (red indicates more stable, blue indicates less stable coexistence, white indicates extinction of one of the sequence pairs). The upper triangle shows the stability measures of the sequences pairs from the lower one (mirrored and rotated ). From the point of view of coexistence two pairs (e.g. -, -) and their reverse (-, -) are not fully equivalent. The reason for this is that the degradation rates are assigned to sequences, always in the same order within a set (this means that the same rates are assigned to e.g. and in the two cases, respectively). Despite this difference the plot is almost symmetrical since degradation rates are taken from a narrow distribution. The parameters are the same as in Fig. 2, for details, see the first section of Text S1. (TIF) [file pcbi.1003193.s001.tif]

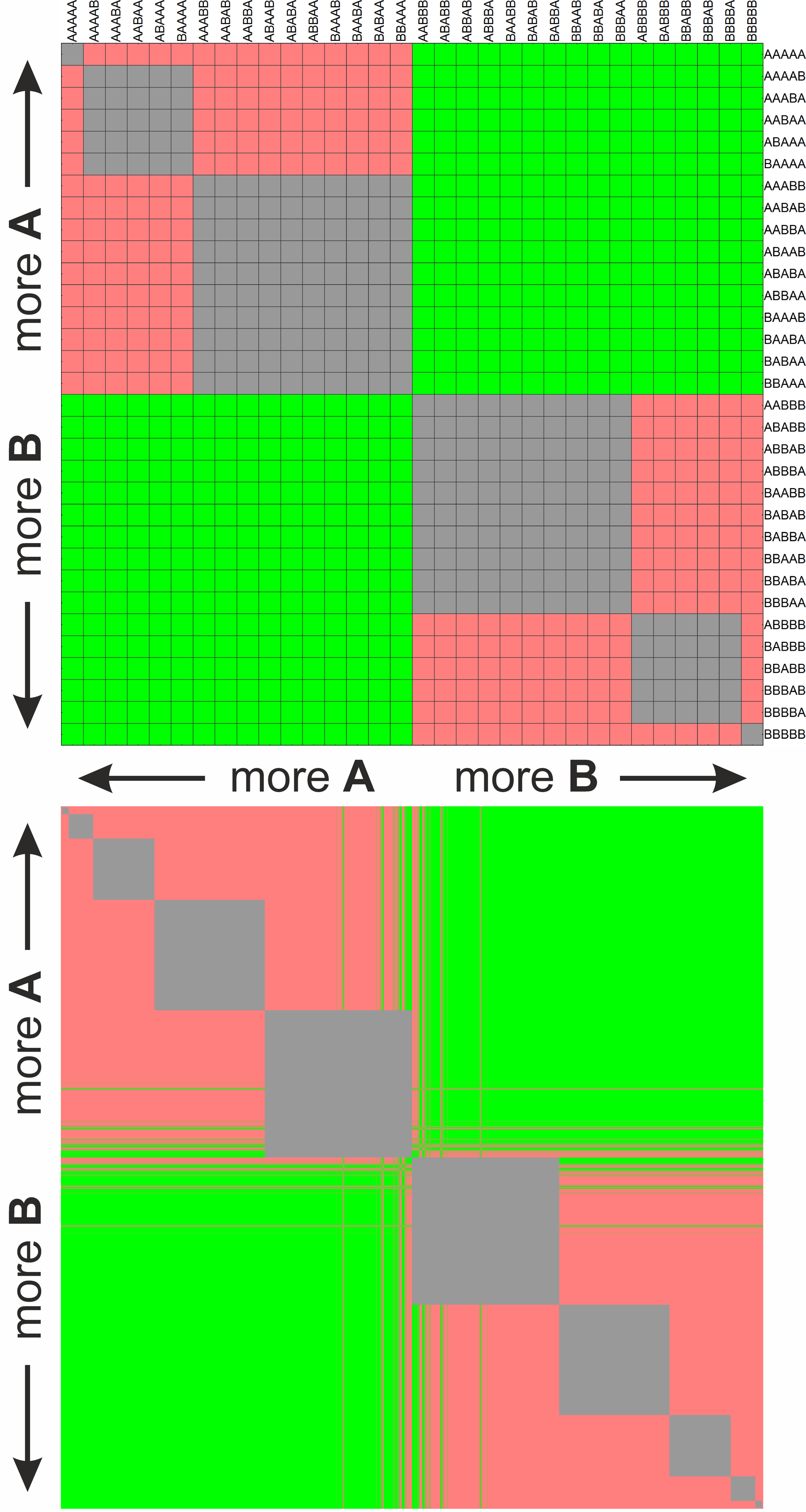

Supplement: Figure S2 — Coexistence plots of pairs of double-stranded sequences of length (upper panel) and (lower panel) using two monomers ( , ) in case of uniform degradation and identical elongation rate constants and non-complementary pairing. The green indicates stable coexistence, grey indicates structurally unstable coexistence, i.e. compositional identity (no coexistence in a biological sense), while pink indicates that there is no coexistence possible. Sequences along the axes are arranged first according to Hamming distance and secondly according to lexicographic ordering from more (top and left) to more (bottom and right). The parameters are the same as in Fig. 4, for details, see the first section of Text S1. (TIF) [file pcbi.1003193.s002.tif]

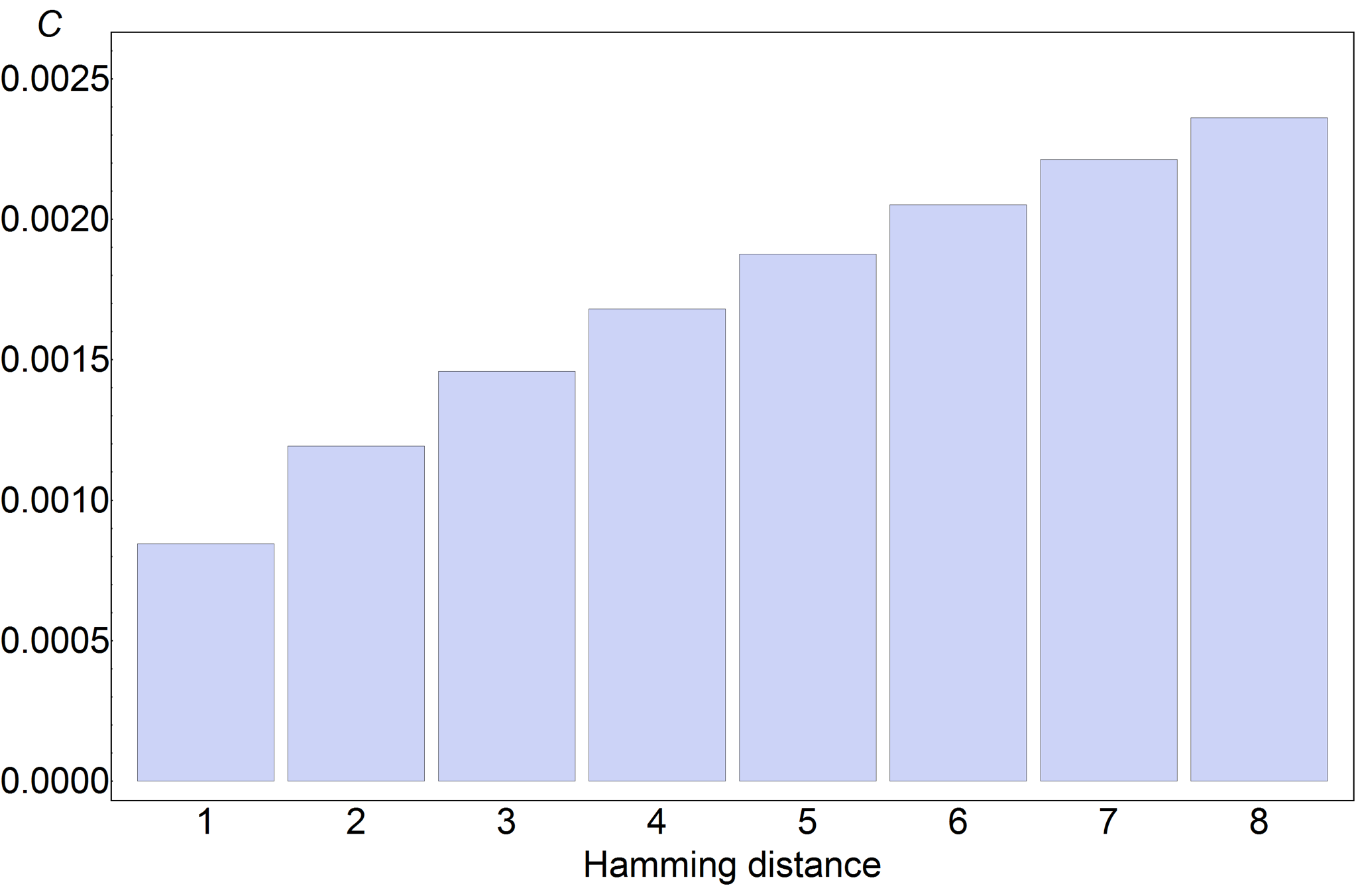

Supplement: Figure S3 — Correlation plot of the fitness landscape as a function of Hamming distances for sequence pairs of length . (TIF) [file pcbi.1003193.s003.tif]
