# Supplementary material for: Gause's Principle and the Effect of Resource Partitioning on the Dynamical Coexistence of Replicating Templates
Source: PLoS Comput Biol. 2013 Aug 22;9(8):e1003193. doi: 10.1371/journal.pcbi.1003193 (PMC3749944; doi:10.1371/journal.pcbi.1003193)
Supplement: Text S1 — Supporting text with sections on 1) Parameters for methods M1, M2, M3 and M4; 2) Analysis of non-complementary pairing with antiparallel polarity; 3) Analysis and analytical results of non-complementary pairing and uniform degradation rates; 4) Proofs; 5) Discussion of the fitness landscape; 6) Examples of coexistence of longer sequences. (PDF) [file pcbi.1003193.s009.pdf]

Supplemetary Information
Gause's principle and the effect of resource partitioning on the dynamical coexistence of replicating templates

András Szilágyi, István Zachar, Eörs Szathmáry

Contents

1 Parameters for methods M1, M2, M3 and M4 **1**
1.1 Parameters for complementary replication . . . 1
1.2 Parameters for non-complementary replication . . . 2

2 Non-complementary pairing with antiparallel polarity **2**

3 Non-complementary pairing and uniform degradation rates: analytical results **2**
3.1 Coexistence on two monomers . . . 3
3.1.1 Criteria for coexistence . . . 5
3.2 Coexistence on four monomers . . . 6

4 Proofs **8**
4.1 Proof of criterion of irregular coexistence . . . 8
4.2 Proof of condition Eq. (38) . . . 9

5 Fitness landscape **10**

6 Examples of coexistence of longer sequences **10**

1 Parameters for methods M1, M2, M3 and M4

1.1 Parameters for complementary replication

For method M1, we used the following parameters (or an appropriate subset of intermediates' degradation rates depending on the number of complementary pairs):

$$P = 1, k_A = k_B = k_C = k_D = 1$$

$$m_A = 0.11;\ m_B = 0.09;\ m_C = 0.12;\ m_D = 0.075.$$

$$d_{x_1} = 0.12;\ d_{x_2} = 0.09;\ d_{x_3} = 0.11;\ d_{x_4} = 0.10$$
$$d_{y_1} = 0.10;\ d_{y_2} = 0.13;\ d_{y_3} = 0.08;\ d_{y_4} = 0.11$$
$$d_{w_1} = 0.08;\ d_{w_2} = 0.13;\ d_{w_3} = 0.11;\ d_{w_4} = 0.09$$
$$d_{z_1} = 0.12;\ d_{z_2} = 0.10;\ d_{z_3} = 0.09;\ d_{z_4} = 0.13$$
$$d_{m_1} = 0.09;\ d_{m_2} = 0.10;\ d_{m_3} = 0.10;\ d_{m_4} = 0.12$$
$$d_{n_1} = 0.08;\ d_{n_2} = 0.12;\ d_{n_3} = 0.11;\ d_{n_4} = 0.09$$
$$d_{q_1} = 0.12;\ d_{q_2} = 0.09\ d_{q_3} = 0.11;\ d_{q_4} = 0.10$$
$$d_{r_1} = 0.07;\ d_{r_2} = 0.12;\ d_{r_3} = 0.09;\ d_{r_4} = 0.11,$$

where the first sequence pair is denoted by x and y, the second is denoted by w and z, etc.

For method M2, we used random degradation rates for intermediates and monomers drawn from the [0.05-0.15] interval with uniform distribution, other parameters (influx P, and elongation rates k_i) were the same as in M1.

1.2 Parameters for non-complementary replication

For method M3, we used the same parameters as in M1 with the following extension of the degradation rates of intermediates x and y (or an appropriate subset):

$$d_{x_5} = 0.12;\ d_{x_6} = 0.13;\ d_{x_7} = 0.10;\ d_{x_8} = 0.08;\ d_{x_9} = 0.11$$
$$d_{y_5} = 0.08;\ d_{y_6} = 0.07;\ d_{y_7} = 0.12;\ d_{y_8} = 0.10;\ d_{y_9} = 0.09.$$

For method M4, we used the same parameters for influx, elongations and monomer degradations as in M1, random degradation rates of intermediates were drawn from the [0.05-0.15] interval with uniform distribution (as in M2).

2 Non-complementary pairing with antiparallel polarity

According to methods M1 and M2 described in the main street, we analyzed the coexistence of different number of pairs of sequences of length four with non-complementary replication and antiparallel polarity. Similar to the case of complementary base pairing, increasing number of template-copy pairs reduces the probability and stability of coexistence. On four different monomers a maximum of four different sequence pairs can coexist. The results of the analysis can be seen in Tables S1 and S2 (methods M1 and M2, respectively; the parameter values used are the same as in the main text, and are listed in the previous section of Text S1). Comparing Tables 1, 2 and Tables S1, S2 (or Fig. 2 and Fig. S1), one can see that the fraction of coexisting pairs and probability of coexistence of pairs with random rate constants are significantly higher in case of non-complementary base pairing. The two strands of a non-complementary pair have the same number of the different monomers (in reverse order) thus a better niche partitioning is possible between the different sequence pairs.

Coexistence can be visualized in case of two coexisting sequence pairs of length L in two dimensions: each cell of a $4^L \cdot 4^L$ matrix represents a certain combination of two pairs, and is labeled by the first sequence of the first pair (rows) and the first sequence of the second pair (columns). Sequences are ordered according to standard lexicographic ordering along the horizontal and vertical axes (see Fig. S1).

We have tested whether long uniform and nonuniform sequence-pairs are able to coexist in case of complementary pairing. In case of uniform sequences, according to our terminology, for 4 bases, there could be only two different sequence-pairs (**AAA**...–**BBB**... and **CCC**...–**DDD**...). These always coexist with any reasonable degradation rate set. (According to Method M2 we have not found such a parameter set for which there is no coexistence.) In case of nonuniform sequences, based on our results, we expect that longer sequences are able to coexist, though statistical analysis of such vast spaces is impossible. For nonuniform sequences, we have tested lengths up to $N = 30$. We have found some examples of coexistence using Method M2, with random search. Found coexisting cases all exhibit linear asymptotic stability. Though these results are far from statistical significance, based only on these successful simulation we found that the decrease in stability is slowing down with incresing chain length. Since these examples exist, it is proven that stable coexistence is possible for longer sequences.

3 Non-complementary pairing and uniform degradation rates: analytical results

In this section we deal with a simplified system. Under the assumption of homologous pairing of monomers and parallel orientation of strands template and copy become identical, thus one replicator implies one sequence only (see Fig. 1 for broader applicability). By further assuming uniform degradation rate constants, we can perform a fully analytic approach.

3.1 Coexistence on two monomers

Given a single-stranded sequence $\mathbf{x_1}$ of length L, using two monomers ($\mathbf{A}$, $\mathbf{B}$), assuming uniform degradation rate, the reaction system is as follows:

$$
\begin{aligned}
\mathbf{w_1} + \mathbf{x_1} &\xrightarrow{k_{\mathbf{w_1}}} \mathbf{x_2} \\
\mathbf{w_2} + \mathbf{x_2} &\xrightarrow{k_{\mathbf{w_2}}} \mathbf{x_3} \\
&\dots \\
\mathbf{w_L} + \mathbf{x_L} &\xrightarrow{k_{\mathbf{w_L}}} 2\mathbf{x_1} \\
&\xrightarrow{P} \mathbf{A}, \mathbf{B} \\
\mathbf{A}, \mathbf{B} &\xrightarrow{m} 0 \\
\mathbf{x_i} &\xrightarrow{d} 0,
\end{aligned}
\tag{1}
$$

where $\mathbf{x_i}$ denotes incomplete double-stranded replication intermediates (for $i > 1$), $\mathbf{w_i} \in \{\mathbf{A}, \mathbf{B}\}$ the type of the ith monomer, and $k_{\mathbf{w_i}} = \{k_A, k_B\}$ the elongation rate constants for the given monomer types at position i ($i = 1 \dots L$). The dynamics of intermediates is as follows (x_i denotes concentration of $\mathbf{x_i}$, w_i denotes concentration of the ith monomer type):

$$\dot{x}_1 = 2k_{\mathbf{w_L}} w_L x_L - \left(k_{\mathbf{w_1}} w_1 + d\right) x_1 \tag{2}$$

$$\dot{x}_i = k_{\mathbf{w_{i-1}}} w_{i-1} x_{i-1} - \left(k_{\mathbf{w_i}} w_i + d\right) x_i \qquad (i = 2 \dots L). \tag{3}$$

It can be deduced from (2), that in equilibrium:

$$\frac{x_1}{x_L} = \frac{2k_{\mathbf{w_L}} w_L}{k_{\mathbf{w_1}} w_1 + d} \tag{4}$$

and from all the equations defined by (3):

$$\frac{x_1}{x_2} \frac{x_2}{x_3} \dots \frac{x_{L-1}}{x_L} = \frac{(k_{\mathbf{w_2}} w_2 + d)(k_{\mathbf{w_3}} w_3 + d) \dots (k_{\mathbf{w_L}} w_L + d)}{k_{\mathbf{w_1}} w_1 k_{\mathbf{w_2}} w_2 \dots k_{\mathbf{w_{L-1}}} w_{L-1}}. \tag{5}$$

As the *lhs*-s of equations (4) and (5) are identical, we get the following identity for the *rhs*-s:

$$2\Pi_{i=1}^{L} k_{\mathbf{w_i}} w_i = \Pi_{i=1}^{L} (k_{\mathbf{w_i}} w_i + d). \tag{6}$$

To investigate coexistence, we introduce a second sequence $\mathbf{y_1}$ of length L. Note that due to homologous pairing of monomers, template and copy are identical, thus a pair of pairs imply only two different sequences (instead of four, as it was for complementary pairing). Intermediates of this new sequence are denoted by $\mathbf{y_i}$ (for $i > 1$), their concentrations by y_i, monomers are denoted by $\mathbf{v_i} \in \{\mathbf{A}, \mathbf{B}\}$, and their concentrations by v_i ($i = 1 \dots L$). The dynamics of the two sequences are similar to (1), thus an analogous method provides the general equation for the monomer concentration:

$$2\Pi_{i=1}^{L} k_{\mathbf{v_i}} v_i = \Pi_{i=1}^{L} (k_{\mathbf{v_i}} v_i + d). \tag{7}$$

Let us denote concentrations of monomers $\mathbf{A}$ and $\mathbf{B}$ as A and B, the number of $\mathbf{A}$-s and $\mathbf{B}$-s in the first sequence as n_1^A and n_1^B, and in the second sequence as n_2^A and n_2^B. Equations (6)-(7) accordingly simplify as:

$$2 = \left(1 + \frac{d}{k_A A}\right)^{n_1^A} \left(1 + \frac{d}{k_B B}\right)^{n_1^B} \tag{8}$$

$$2 = \left(1 + \frac{d}{k_A A}\right)^{n_2^A} \left(1 + \frac{d}{k_B B}\right)^{n_2^B}. \tag{9}$$

The equations for equilibrium monomer-concentration (8)-(9) only have unique solutions if $n_1^A \neq n_2^A$, i.e. there is a different amount of **A** in the two sequences (and consequently, different amount of **B** as well). $n_1^A = n_2^A$ is the definition of *compositional identity* [1]. Relying on that $n_i^A + n_i^B = L$, the equilibrium monomer concentrations are (given that $\rho = \sqrt[L]{2}$):

$$\hat{A} = \frac{d}{(\rho-1)k_A}; \quad \hat{B} = \frac{d}{(\rho-1)k_B} \qquad \text{(if there is no compositional identity!)} \tag{10}$$

In light of the concentrations, let us investigate the dynamics. Note, that:

$$k_{\mathbf{w_i}} w_i = \frac{1}{\rho - 1} d \tag{11}$$

$$k_{\mathbf{w_i}} w_i + d = \frac{\rho}{\rho - 1} d. \tag{12}$$

Substituting these into Eqs. (2)-(3) ($d \neq 0$):

$$\frac{x_{i-1}}{x_i} = \rho \qquad (i = 2 \dots L) \tag{13}$$

$$\frac{x_L}{x_1} = \frac{1}{2}\rho. \tag{14}$$

Similar results can be deduced for the intermediates of the second sequence y_i. For further advancement, one has to calculate the dynamics of the generation of monomers. Based on (1), we can formally write the following:

$$\dot{A} = P - k_A A \sum_{i=1}^{L} (\delta_{\mathbf{w_i},\mathbf{A}} x_i + \delta_{\mathbf{v_i},\mathbf{A}} y_i) - m_A A \tag{15}$$

$$\dot{B} = P - k_B B \sum_{i=1}^{L} (\delta_{\mathbf{w_i},\mathbf{B}} x_i + \delta_{\mathbf{v_i},\mathbf{B}} y_i) - m_B B, \tag{16}$$

where $\delta_{i,j} = 1$ if $i = j$, otherwise 0 (Kronecker delta), and m is the degradation rate of the monomers. Since Eqs. (13) and (14) give the ratio of concentrations of "neighboring" intermediates, the concentration of each intermediate can be expressed by using the last (L^{th}) concentration:

$$x_i = \rho^{L-i} x_L, \quad y_i = \rho^{L-i} y_L \qquad (i = 1 \dots L). \tag{17}$$

By substituting these into Eqs. (15) and (16), we get a set of *linear* equations of two variables, defining the equilibrium values of $\hat{x}_L$ and $\hat{y}_L$:

$$0 = k_A \hat{A} \sum_{i=1}^{L} \left(\delta_{\mathbf{w_i},\mathbf{A}} \rho^{L-i} \hat{x}_L + \delta_{\mathbf{v_i},\mathbf{A}} \rho^{L-i} \hat{y}_L \right) + m_A \hat{A} - P \tag{18}$$

$$0 = k_B \hat{B} \sum_{i=1}^{L} \left(\delta_{\mathbf{w_i},\mathbf{B}} \rho^{L-i} \hat{x}_L + \delta_{\mathbf{v_i},\mathbf{B}} \rho^{L-i} \hat{y}_L \right) + m_B \hat{B} - P. \tag{19}$$

For example in case of the coexistence of **BABA**–**AAAB** ($\mathbf{w_2} = \mathbf{w_4} = \mathbf{A},\ \mathbf{w_1} = \mathbf{w_3} = \mathbf{B},\ \mathbf{v_1} = \mathbf{v_2} = \mathbf{v_3} = \mathbf{A},\ \mathbf{v_4} = \mathbf{B}$):

$$0 = k_A \hat{A} \left[(\rho^2 + 1)\hat{x}_L + (\rho^3 + \rho^2 + \rho)\hat{y}_L \right] + m_A \hat{A} - P \tag{20}$$

$$0 = k_B \hat{B} \left[(\rho^3 + \rho)\hat{x}_L + \hat{y}_L \right] + m_B \hat{B} - P. \tag{21}$$

[1] In case of compositional identity, the equilibrium concentrations of monomers A and B are underdetermined, only the ratio $\hat{A}/\hat{B}$ is given.

For the positivity test, we introduce the following notation for the constants of the power sum of ρ:

$$W^A = \sum_{i=1}^{L} \delta_{\mathbf{w_i},\mathbf{A}} \rho^{L-i}, \quad W^B = \sum_{i=1}^{L} \delta_{\mathbf{w_i},\mathbf{B}} \rho^{L-i} \tag{22}$$

$$V^A = \sum_{i=1}^{L} \delta_{\mathbf{v_i},\mathbf{A}} \rho^{L-i}, \quad V^B = \sum_{i=1}^{L} \delta_{\mathbf{v_i},\mathbf{B}} \rho^{L-i}. \tag{23}$$

With the notation introduced above, the solution of the system of Eqs. (18)-(19) provides the concentrations of the last intermediates of $\mathbf{x}$ and $\mathbf{y}$:

$$\hat{x}_L = \frac{\begin{vmatrix} \frac{P - m_A \hat{A}}{k_A \hat{A}} & \frac{P - m_B \hat{B}}{k_B \hat{B}} \\ V^A & V^B \end{vmatrix}}{\begin{vmatrix} W^A & W^B \\ V^A & V^B \end{vmatrix}} = \frac{\frac{P - m_A \hat{A}}{k_A \hat{A}} V^B - \frac{P - m_B \hat{B}}{k_B \hat{B}} V^A}{W^A V^B - W^B V^A} \tag{24}$$

$$\hat{y}_L = \frac{\begin{vmatrix} W^A & W^B \\ \frac{P - m_A \hat{A}}{k_A \hat{A}} & \frac{P - m_B \hat{B}}{k_B \hat{B}} \end{vmatrix}}{\begin{vmatrix} W^A & W^B \\ V^A & V^B \end{vmatrix}} = \frac{\frac{P - m_B \hat{B}}{k_B \hat{B}} W^A - \frac{P - m_A \hat{A}}{k_A \hat{A}} W^B}{W^A V^B - W^B V^A}, \tag{25}$$

where $\hat{A}$ and $\hat{B}$ depend on parameters according to Eq. (10). A more concise determinant-formalism will be used later on when we assume more than two monomers.

3.1.1 Criteria for coexistence

Let us assume that influx can counter degradation:

$$P > m_A \hat{A}, \quad P > m_B \hat{B} \longrightarrow P > \frac{d m_A}{(\rho - 1) k_A}, \quad P > \frac{d m_B}{(\rho - 1) k_B}. \tag{26}$$

Based on the equilibrium concentrations, the positivity criteria for $\hat{x}_L$ are ($Q = \frac{P - m_A \hat{A}}{P - m_B \hat{B}} \frac{k_B \hat{B}}{k_A \hat{A}}$):

$$\frac{V^A}{V^B} \lesseqgtr Q \quad \text{and} \quad \frac{V^A}{V^B} \lesseqgtr \frac{W^A}{W^B}, \tag{27}$$

similarly, $\hat{y}_L$ is positive if:

$$\frac{W^A}{W^B} \gtreqless Q \quad \text{and} \quad \frac{V^A}{V^B} \lesseqgtr \frac{W^A}{W^B}. \tag{28}$$

According to the above equations, the criteria of coexistence are:

$$V^A \lesseqgtr Q V^B \quad \text{and} \quad W^A \gtreqless Q W^B. \tag{29}$$

To sum up, coexistence is possible if $V^A - QV^B$ and $W^A - QW^B$ are of *different signs.*

If the two elongation rate constants are identical ($k_A = k_B$) the parameter $Q = 1$, thus the simple criteria of coexistence are the following:

$$V^A \lesseqgtr V^B, \quad \text{and} \quad W^A \gtreqless W^B. \tag{30}$$

For example, given the sequences **BABA** and **AAAB** ($L = 4$) and $k_A = k_B$:

$$W^A = \rho^2 + 1, \quad W^B = \rho^3 + \rho, \quad V^A = \rho^3 + \rho^2 + \rho, \quad V^B = 1. \tag{31}$$

Since $V^A > V^B$ and $W^A < W^B$, coexistence is *present*. Though in case of **ABAA** and **AABB**:

$$W^A = \rho^3 + \rho + 1, \quad W^B = \rho^2, \quad V^A = \rho^3 + \rho^2, \quad V^B = \rho + 1. \tag{32}$$

Since $V^A > V^B$ and $W^A > W^B$, coexistence is *not possible*!

3.2 Coexistence on four monomers

In this section we extend the formalism used so far and reformulate the dynamics introduced in the previous section to four monomers **A**, **B**, **C**, **D**. First, we modify the notation by introducing the upper index j describing different sequences. The descriptor of the ith monomer in the jth sequence is $\mathbf{w_i^j} \in \{\mathbf{A}, \mathbf{B}, \mathbf{C}, \mathbf{D}\}$ and its concentration is w_i^j. Similarly, the descriptor of the intermediates and their corresponding concentrations are $\mathbf{x_i^j}$ and x_i^j, respectively. The reaction-scheme of the jth sequence is (cf. Eq. (1))

$$\begin{aligned}
\mathbf{w_1^j} + \mathbf{x_1^j} &\xrightarrow{k_{\mathbf{w_1^j}}} \mathbf{x_2^j} \\
\mathbf{w_2^j} + \mathbf{x_2^j} &\xrightarrow{k_{\mathbf{w_2^j}}} \mathbf{x_3^j} \\
&\cdots \\
\mathbf{w_L^j} + \mathbf{x_L^j} &\xrightarrow{k_{\mathbf{w_L^j}}} 2\mathbf{x_1^j} \\
&\xrightarrow{P} \mathbf{A}, \mathbf{B}, \mathbf{C}, \mathbf{D} \\
\mathbf{A}, \mathbf{B}, \mathbf{C}, \mathbf{D} &\xrightarrow{m} 0 \\
\mathbf{x_i^j}, \mathbf{y_i^j} &\xrightarrow{d} 0,
\end{aligned} \tag{33}$$

where $k_{\mathbf{w_i^j}} \in \{k_A, k_B, k_C, k_D\}$ is the elongation rate constant corresponding to monomer $\mathbf{w_i^j}$. The dynamics of the intermediates of sequence j is (cf. Eqs. (2)-(3))

$$\dot{x}_1^j = 2k_{\mathbf{w_L^j}} w_L^j x_L^j - \left(k_{\mathbf{w_1^j}} w_1^j + d\right) x_1^j \tag{34}$$

$$\dot{x}_i^j = k_{\mathbf{w_{i-1}^j}} w_{i-1}^j x_{i-1}^j - \left(k_{\mathbf{w_i^j}} w_i^j + d\right) x_i^j \qquad (i = 2 \dots L). \tag{35}$$

By calculating the ratio x_1^j / x_L^j from Eq. (34) and from Eq. (35) we get the following formula (see Eqs. (4)-(5)):

$$2\Pi_{i=1}^L k_{\mathbf{w_i^j}} w_i^j = \Pi_{i=1}^L (k_{\mathbf{w_i^j}} w_i^j + d). \tag{36}$$

We denote the number of monomer **A** in the first sequence by n_1^A, etc. In case of coexistence of four sequences, we have (cf. Eqs. (8)-(9)):

$$2 = \left(1 + \frac{d}{k_A A}\right)^{n_j^A} \left(1 + \frac{d}{k_B B}\right)^{n_j^B} \cdot \left(1 + \frac{d}{k_C C}\right)^{n_j^C} \left(1 + \frac{d}{k_D D}\right)^{n_j^D}, \quad (j = 1 \dots 4). \tag{37}$$

These four equations ($j = 1 \dots 4$) have a unique solution iff

$$\Delta = \begin{vmatrix} n_1^A & n_2^A & n_3^A & n_4^A \\ n_1^B & n_2^B & n_3^B & n_4^B \\ n_1^C & n_2^C & n_3^C & n_4^C \\ n_1^D & n_2^D & n_3^D & n_4^D \end{vmatrix} \neq 0. \tag{38}$$

In this case, the equilibrium concentrations of the monomers are

$$\hat{A} = \frac{d}{(\rho-1)k_A}; \quad \hat{B} = \frac{d}{(\rho-1)k_B} \tag{39}$$

$$\hat{C} = \frac{d}{(\rho-1)k_C}; \quad \hat{D} = \frac{d}{(\rho-1)k_D}, \tag{40}$$

for details and proof, see Eqs. (8)-(10) and the next section. Using these equilibrium values of monomer concentrations, it is easy to see that

$$k_{\mathbf{w_i^j}} w_i^j = \frac{1}{\rho-1} d, \tag{41}$$

$$k_{\mathbf{w_i^j}} w_i^j + d = \frac{\rho}{\rho-1} d. \tag{42}$$

Back-substituting the results into Eqs. (34) and (35), the ratios of the concentration of neighboring intermediates are ($i = 2 \ldots L$):

$$\frac{x_{i-1}^j}{x_i^j} = \rho, \tag{43}$$

$$\frac{x_L^j}{x_1^j} = \frac{1}{2}\rho. \tag{44}$$

Consequently, concentrations of all intermediates can be expressed by the intermediate concentration of the last step (x_L^j) as:

$$x_i^j = \rho^{L-i} x_L^j. \tag{45}$$

From the reaction scheme (33), the dynamics of monomers has a compact form (cf. Eqs. (15)-(16))

$$\dot{A} = P - k_A A \sum_{i=1}^{L} \sum_{j=1}^{4} \delta_{\mathbf{w_i^j},\mathbf{A}} x_i^j - m_A A \tag{46}$$

$$\dot{B} = P - k_B B \sum_{i=1}^{L} \sum_{j=1}^{4} \delta_{\mathbf{w_i^j},\mathbf{B}} x_i^j - m_B B \tag{47}$$

$$\dot{C} = P - k_C C \sum_{i=1}^{L} \sum_{j=1}^{4} \delta_{\mathbf{w_i^j},\mathbf{C}} x_i^j - m_C C \tag{48}$$

$$\dot{D} = P - k_D D \sum_{i=1}^{L} \sum_{j=1}^{4} \delta_{\mathbf{w_i^j},\mathbf{D}} x_i^j - m_D D, \tag{49}$$

where $\mathbf{R} \in \{\mathbf{A}, \mathbf{B}, \mathbf{C}, \mathbf{D}\}$ and $\delta_{\mathbf{w_i^j},\mathbf{R}}$ is 1 if $\mathbf{w_i^j}$ is $\mathbf{R}$, and zero otherwise (Kronecker delta) and m indicates monomer degradation rate. We introduce an abbreviation similar to Eqs. (22)-(23):

$$W_j^R = \sum_{i=1}^{L} \delta_{\mathbf{w_i^j},\mathbf{R}} \rho^{L-i}. \tag{50}$$

For example, if the first sequence ($j = 1$) is **ABBCDAAC** the W's have the following formulae:

$$W_1^A = \rho^7 + \rho^2 + \rho, \quad W_1^B = \rho^6 + \rho^5, \quad W_1^C = \rho^4 + 1, \quad W_1^D = \rho^3, \tag{51}$$

where $\rho = \sqrt[8]{2}$ and $L = 8$.

As one can prove (e.g. by direct back-substitution), the equilibrium concentrations of the last step of the intermediate of sequences 1, 2, 3, and 4 are (note that the superscript of x is the sequence number is not a power!):

$$x_L^1 = \frac{1}{H}\begin{vmatrix} \left(\frac{P-m_A\hat{A}}{k_A\hat{A}}\right) & \left(\frac{P-m_B\hat{B}}{k_B\hat{B}}\right) & \left(\frac{P-m_C\hat{C}}{k_C\hat{C}}\right) & \left(\frac{P-m_D\hat{D}}{k_D\hat{D}}\right) \\ W_1^B & W_2^B & W_3^B & W_4^B \\ W_1^C & W_2^C & W_3^C & W_4^C \\ W_1^D & W_2^D & W_3^D & W_4^D \end{vmatrix} \tag{52}$$

$$x_L^2 = \frac{1}{H}\begin{vmatrix} W_1^A & W_2^A & W_3^A & W_4^A \\ \left(\frac{P-m_A\hat{A}}{k_A\hat{A}}\right) & \left(\frac{P-m_B\hat{B}}{k_B\hat{B}}\right) & \left(\frac{P-m_C\hat{C}}{k_C\hat{C}}\right) & \left(\frac{P-m_D\hat{D}}{k_D\hat{D}}\right) \\ W_1^C & W_2^C & W_3^C & W_4^C \\ W_1^D & W_2^D & W_3^D & W_4^D \end{vmatrix} \tag{53}$$

$$x_L^3 = \frac{1}{H}\begin{vmatrix} W_1^A & W_2^A & W_3^A & W_4^A \\ W_1^B & W_2^B & W_3^B & W_4^B \\ \left(\frac{P-m_A\hat{A}}{k_A\hat{A}}\right) & \left(\frac{P-m_B\hat{B}}{k_B\hat{B}}\right) & \left(\frac{P-m_C\hat{C}}{k_C\hat{C}}\right) & \left(\frac{P-m_D\hat{D}}{k_D\hat{D}}\right) \\ W_1^D & W_2^D & W_3^D & W_4^D \end{vmatrix} \tag{54}$$

$$x_L^4 = \frac{1}{H}\begin{vmatrix} W_1^A & W_2^A & W_3^A & W_4^A \\ W_1^B & W_2^B & W_3^B & W_4^B \\ W_1^C & W_2^C & W_3^C & W_4^C \\ \left(\frac{P-m_A\hat{A}}{k_A\hat{A}}\right) & \left(\frac{P-m_B\hat{B}}{k_B\hat{B}}\right) & \left(\frac{P-m_C\hat{C}}{k_C\hat{C}}\right) & \left(\frac{P-m_D\hat{D}}{k_D\hat{D}}\right) \end{vmatrix}, \tag{55}$$

where

$$H = \begin{vmatrix} W_1^A & W_2^A & W_3^A & W_4^A \\ W_1^B & W_2^B & W_3^B & W_4^B \\ W_1^C & W_2^C & W_3^C & W_4^C \\ W_1^D & W_2^D & W_3^D & W_4^D \end{vmatrix}. \tag{56}$$

The four sequences can coexist if x_1, x_2, x_3 and x_4 concentrations are all positive. From Eq. (37) it is straightforward to conclude that only a maximum of four sequences can coexist on four monomers. Numerical investigations over the range of $L = 3 \ldots 6$ (15000 independent runs for each L with random parameters) have confirmed that five sequences can not coexist. In case of identical degradation rates, the coexistence of five sequences is structurally unstable.

In the range of $L = 3 \ldots 6$ (15000 independent runs for each L with random parameters) we have confirmed that in case of four monomers (if the coexistence is locally asymptotically stable) the numerically and analytically derived monomer concentrations are the same.

4 Proofs

4.1 Proof of criterion of irregular coexistence

According to Eq. (29), the criterion of the coexistence of two sequences is (we only use the relation of one direction, the other can be devised by swapping $\mathbf{A} \leftrightarrow \mathbf{B}$):

$$W^A > W^B \quad \text{and} \quad V^A < V^B. \tag{57}$$

It can be assumed that if a sequence (e.g. $\mathbf{W}$) has for example more $\mathbf{A}$ than $\mathbf{B}$ then $W^A > W^B$. Let's investigate how this principle can be violated. For sake of simplicity, let us take a sequence that has x **A**-s in its „head" and $L - x$ **B**-s in its „tail". In this case

$$W^A = \rho^{L-1} + \rho^{L-2} + \cdots + \rho^{L-x} \tag{58}$$
$$W^B = \rho^{L-x-1} + \cdots + \rho + 1. \tag{59}$$

The question is: what values of x yield $W^A > W^B$? Using the sum formula of the geometric progression:

$$W^B = \frac{\rho^{L-x} - 1}{\rho - 1} \tag{60}$$
$$W^A = \frac{\rho^L - 1}{\rho - 1} - W^B = \frac{2 - \rho^{L-x}}{\rho - 1}, \tag{61}$$

where we used $\rho^L = 2$. After simplification: $W^A > W^B$ if

$$x > \frac{\ln 4 - \ln 3}{\ln 2} L \approx 0,415L. \tag{62}$$

Thus if there is a continuous, homogeneous subsequence (containing one monomer only) of at least length $\lceil 0,415L \rceil$ in the *head* of the sequence then this sequence dynamically *behaves* (concerning coexistence) as if it had this monomer in majority. This behavior can be the result of a non-homogeneous subsequence in the head *if* the sequence length is large enough.

4.2 Proof of condition Eq. (38)

The solution of the system of Eq. (37) is:

$$\hat{A} = \frac{d}{k_A} \left[2^{\frac{\Delta_A}{\Delta}} - 1 \right]^{-1} \tag{63}$$
$$\hat{B} = \frac{d}{k_B} \left[2^{\frac{\Delta_B}{\Delta}} - 1 \right]^{-1} \tag{64}$$
$$\hat{C} = \frac{d}{k_C} \left[2^{\frac{\Delta_C}{\Delta}} - 1 \right]^{-1} \tag{65}$$
$$\hat{D} = \frac{d}{k_D} \left[2^{\frac{\Delta_D}{\Delta}} - 1 \right]^{-1}, \tag{66}$$

where

$$\Delta_A = \begin{vmatrix} 1 & 1 & 1 & 1 \\ n_1^B & n_2^B & n_3^B & n_3^B \\ n_1^C & n_2^C & n_3^C & n_3^C \\ n_1^D & n_2^D & n_3^D & n_3^D \end{vmatrix}, \quad \Delta_B = \begin{vmatrix} n_1^A & n_2^A & n_3^A & n_4^A \\ 1 & 1 & 1 & 1 \\ n_1^C & n_2^C & n_3^C & n_4^C \\ n_1^D & n_2^D & n_3^D & n_3^D \end{vmatrix} \tag{67}$$

$$\Delta_C = \begin{vmatrix} n_1^A & n_2^A & n_3^A & n_4^A \\ n_1^B & n_2^B & n_3^B & n_4^B \\ 1 & 1 & 1 & 1 \\ n_1^D & n_2^D & n_3^D & n_4^D \end{vmatrix}, \quad \Delta_D = \begin{vmatrix} n_1^A & n_2^A & n_3^A & n_4^A \\ n_1^B & n_2^B & n_3^B & n_4^B \\ n_1^C & n_2^C & n_3^C & n_4^C \\ 1 & 1 & 1 & 1 \end{vmatrix} \tag{68}$$

and

$$\Delta = \begin{vmatrix} n_1^A & n_2^A & n_3^A & n_4^A \\ n_1^B & n_2^B & n_3^B & n_4^B \\ n_1^C & n_2^C & n_3^C & n_4^C \\ n_1^D & n_2^D & n_3^D & n_4^D \end{vmatrix}. \tag{69}$$

As the total number of monomers is L, we get

$$n_i^D = L - n_i^A - n_i^B - n_i^C, \qquad (i = 1, 2, 3, 4)$$

and from this it is easy to see that

$$\Delta_A = \Delta_B = \Delta_C = \Delta_S = \frac{1}{L}\Delta.$$

Thus there is no unique solution if $\Delta_i = 0$.

5 Fitness landscape

The fitness (ϕ_{x_i}) of the sequence pair (defined by the first strand x_i) is the leading eigenvalue of the dynamical matrix of the system of complementary pairing, four bases and antiparallel strand polarity. We assume a non-regulated system as the resource concentrations are fixed at a constant value. For decay rates, see the first section of Text S1, elongation rates are $k_A = 1.12$, $k_B = 0.95$, $k_C = 0.86$, $k_D = 1.47$ after Deck et al. ([1], Table S1), multiplied by 10, assuming enzyme-free elongation and $\mathbf{A} \sim A$, $\mathbf{B} \sim U$, $\mathbf{C} \sim C$ and $\mathbf{D} \sim G$ monomer correspondence with RNA bases. We have measured the fitness difference of all possible sequence pairs (x_i, x_j) with Hamming distance $HD(x_i, x_j) = d$, $(d = 1 \ldots L)$. The number of such pairs for a given d is denoted as N_d, the fitness of a sequence is denoted by ϕ_{x_i}. The calculated differences were normalized, according to the formula:

$$C_d = \sqrt{\sum_{(x_i,x_j)\in HD(x_i,x_j)=d} \frac{\left(\phi_{x_i} - \phi_{x_j}\right)^2}{N_d}} \tag{70}$$

yielding the normalized average correlation value for a given Hamming distance d (see Fig. S3).

6 Examples of coexistence of longer sequences

We have tested whether long non-uniform sequences are able to coexist (assuming complementary strand pairing). For each sequence-pair group (4 pairs) we have generated a set of degradation rates for all components with uniform distribution from the [0.05, 0.15] interval, as in Method M2. As the full combined sequence space for such lengths is enormous, we have not performed exhaustive search for coexisting cases. For the different lengths we have found a few examples of coexistence with brute force search (see Tables S3, S4 and S5 for $L = 20, 25$ and 30, respectively). Our results show that 8 sequences (4 sequence pairs) of length up to $L = 30$ can stably coexist (linear asymptotic stability was explicitly tested). Since these examples exist it is proven that stable coexistence is possible for longer sequence lengths. Based on these results, we expect that the trend is true for higher sequence lengths as well, though statistical analysis of such vast spaces is not possible. Though we have demonstrated the gradual decrease of stability as a function of the growing sequence length, we do not dare to extrapolate from these data to much longer sequences.

References

1. Christopher D, Jauker M, Richert C (2011) Efficient enzyme-free copying of all four nucleobases templated by immobilized RNA. Nature Chemistry 3: 603-608.
